# Synthesis of guanidinium–sulfonimide ion pairs: towards novel ionic liquid crystals

**DOI:** 10.3762/bjoc.9.121

**Published:** 2013-06-05

**Authors:** Martin Butschies, Manuel M Neidhardt, Markus Mansueto, Sabine Laschat, Stefan Tussetschläger

**Affiliations:** 1 Institut für Organische Chemie, Universität Stuttgart, Pfaffenwalring 55, 70569 Stuttgart, Germany

**Keywords:** anion exchange, ionic liquid crystals, ion pairs, mesophases, sulfonimides

## Abstract

The recently introduced concept of ionic liquid crystals (ILCs) with complementary ion pairs, consisting of both, mesogenic cation and anion, was extended from guanidinium sulfonates to guanidinium sulfonimides. In this preliminary study, the synthesis and mesomorphic properties of selected derivatives were described, which provide the first example of an ILC with the sulfonimide anion directly attached to the mesogenic unit.

## Introduction

**Scheme 1:**
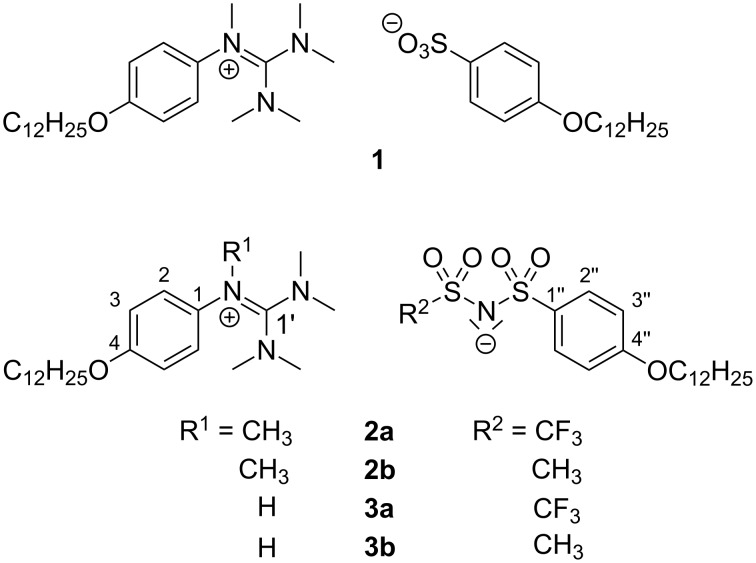
Complementary guanidinium sulfonate **1** and guanidinium–sulfonimide ion pairs **2** and **3**.

While ionic liquids, i.e., molten salts composed of either organic cation or anion (or both) with melting points far below 100 °C, are extensively used as designer solvents, electrolytes for lithium ion batteries, dye-sensitized solar cells, and the electrochemical deposition of metals [1–5], the corresponding ionic liquids with thermotropic liquid-crystalline properties, i.e., ILCs, are a much younger class of compounds [6–8]. Although Heintz was the pioneer, who observed in 1854 melting and clearing transitions upon heating of magnesium myristate [9–10], he did not recognize this as liquid-crystalline behavior. The first regular pyridinium ILCs were reported in 1938 by Knight and Shaw [11–12], followed by seminal findings by Seddon and Bruce [13]. Regarding ionic liquids, sulfonimides have been used in various ways. Particularly interesting is the symmetrical bistriflimide anion [14], which leads to desirable properties such as hydrolytic stability, low viscosity or low melting points in the ionic liquids [1,4,15–19]. By using an elongated perfluoroalkyl chain at the bistriflimide anion in combination with short chain-substituted pyrrolidinium cations MacFarlane was able to induce plastic crystal phases and liquid-crystalline phases at room temperature [20]. Ion pairs consisting of perfluorosulfonylimide anions and imidazolium cations with short alkyl chains were studied by DesMarteau resulting in room-temperature ionic liquids [21]. In contrast, ILCs with bistriflimide anions are much less explored, because the sterically demanding anion often inhibits the formation of a mesophase [6,22]. Liquid-crystalline phases were found for viologen salts [23–28], imidazolium ILCs [29–31], pyrrolidinium ILCs [32–33] and ionic polymers [34–37]. Sulfonimides, which are directly bound to a mesogenic group, have not been described until now. We have recently described the concept of complementary ion pairs, where guanidinium sulfonate **1** with both mesogenic cation and anion displayed improved mesophase stability and increased phase widths as compared to their counterparts bearing a nonmesogenic spherical halide counterion [38–39] (Scheme 1).

We were thus interested, whether this concept could be also used to generate the corresponding sulfonimide ion pairs **2** and **3** with mesomorphic properties. The results of this preliminary study are discussed below.

## Results and Discussion

**Scheme 2:**
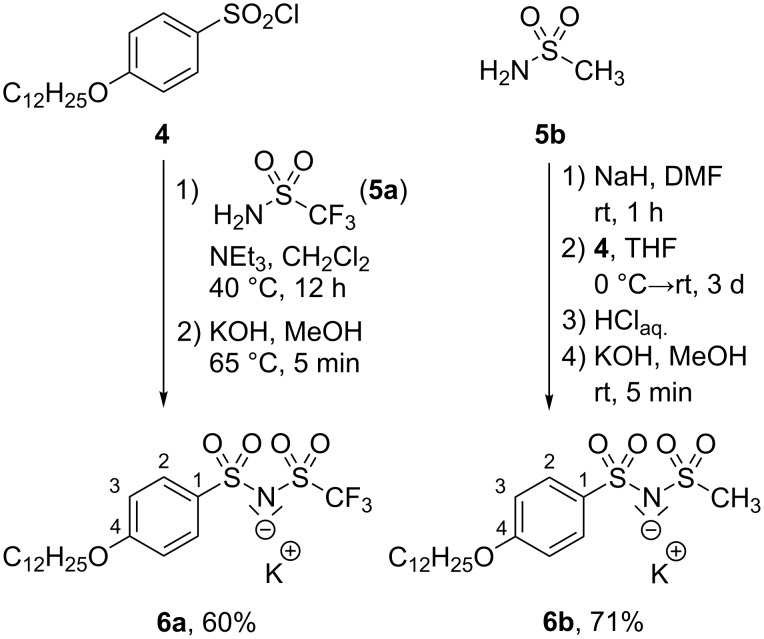
Synthesis of the potassium sulfonimides **6a** and **6b**.

The synthesis of guanidinium–sulfonimide ion pairs commenced with the commercially available sulfonyl chloride **4** [38], which was treated with trifluoromethanesulfonamide (**5a**) in the presence of NEt_3_ following a procedure by Hesemann and Brunel [40]. Then the fluorinated sulfonimide was converted to the potassium salt **6a** after recrystallization from MeOH in 60% yield (Scheme 2).

The corresponding nonfluorinated sulfonimide K^+^-salt **6b** was obtained by deprotonation of methanesulfonamide (**5b**) with NaH followed by treatment with sulfonyl chloride **4** according to a method by Dick and Townsend [41]. Analogous deprotonation with KOH yielded **6b** in 71%.

The K^+^-salts were prepared due to their more convenient isolation as compared to the corresponding protonated compounds. However, the K^+^-sulfonimides **6a**,**b** were not used for a direct methyl transfer towards the synthesis of the desired guanidinium–sulfonimide ion pairs in a similar way that the arylsulfonic acid methylesters were previously used as methyl transfer reagents [38], because we wanted to avoid the activation with dimethyl sulfate reported by DesMarteau [21]. Therefore we planned an indirect formation of the ion pairs by anion exchange via salt metathesis. In order to be successful, two requirements have to be met. First, the solubility of the sulfonimide salts **6a**,**b** in the solvent must be sufficient. Second, one of the products must be insoluble in order to shift the equilibrium towards complete conversion. In contrast to the sodium 4-alkoxyphenylsulfonates both potassium salts **6a**,**b** are soluble in boiling MeCN, so that both conditions for a successful salt metathesis are fulfilled.

**Scheme 3:**
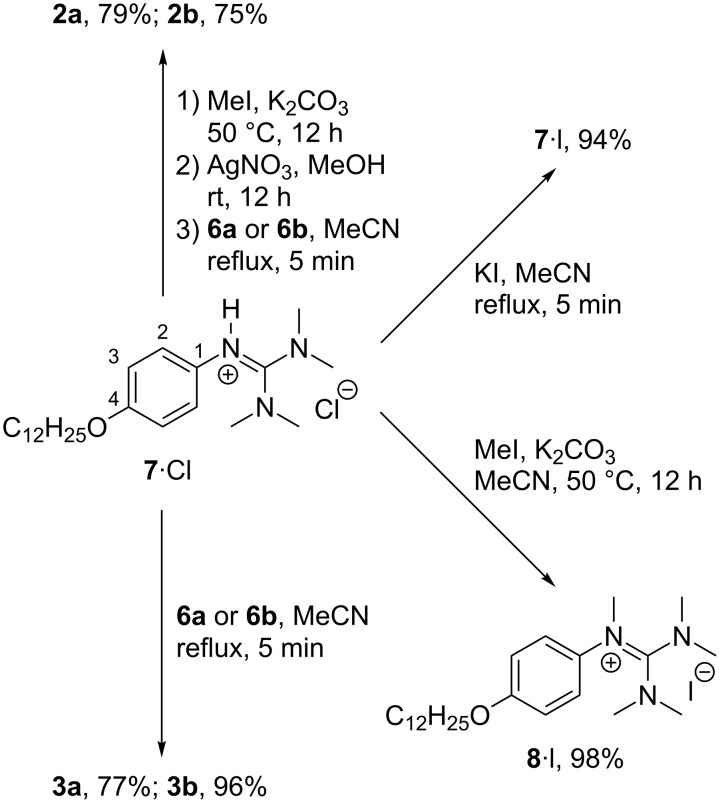
Synthesis of guanidinium sulfonimides **2a**,**b**, **3a**,**b** and iodides **7∙I**, **8∙I**.

The known guanidinium chloride **7**∙Cl [42] was treated with MeI in the presence of K_2_CO_3_ to afford the N-methylated guanidinium iodide **8**∙I (Scheme 3) [38–39].

However, this intermediate did not allow a salt metathesis, because the resulting KI is highly soluble in MeCN (and other organic solvents). Therefore, the intermediate was treated with AgNO_3_ in MeOH. The resulting N-methylated guanidinium nitrate was then reacted with **6a** or **6b** in MeCN to the desired ion pairs **2a** and **2b** in 79% and 75% yield, respectively, while the precipitating KNO_3_ shifted the salt metathesis to completion (Scheme 3).

The good solubility of the K^+^-sulfonimide salts **6a**,**b** in MeCN was further used for a salt metathesis towards the N-protonated guanidinium–sulfonimide ion pairs **3a**,**b** by heating **7**∙Cl with **6a** or **6b** under reflux. The resulting ion pairs **3a** and **3b** were isolated in 77% and 96% yield, respectively. In comparison the guanidinium iodide **7**∙I was obtained in 94% yield by heating **7**∙Cl with KI in MeCN under reflux (Scheme 3). The mesomorphic properties of ion pairs **2**,**3** and guanidinium halides **7**∙I, **7**∙Cl, **8**∙I were studied by differential scanning calorimetry (DSC) and polarizing optical microscopy (POM). The results of the DSC experiments are summarized in Table 1.

**Table 1:** Phase-transition temperatures (°C) and enthalpies (kJ mol^−1^) of guanidinium–sulfonimide ion pairs **2** and **3** and the corresponding guanidinium salts **7**∙I, **7**∙Cl and **8**∙I^a^.

Entry	Compound	Phase transitions (onset (°C)) and transition enthalpies (given in parentheses) (kJ mol^−1^) upon second heating

1	**2a**	Cr 61 (31.8) I^b^
2	**2b**	Cr 93 (59.1) I
3	**8**∙I	Cr_1_ 49 (8.4) Cr_2_ 117 (25.8) I^c^
4	**3a**	Cr_1_ 8 (18.6) Cr_2_ 19 (0.8) Cr_3_ 37 (−44.9) Cr_4_ 75 (48.6) I
5	**3b**	Cr_1_ 5 (8.1) Cr_2_ 27 (−54.4) Cr_3_ 71 (73.1) SmA 87 (1.4) I
6	**7**∙I	Cr_1_ 55 (−44.3) Cr_2_ 130 (37.2) I
7	**7**∙Cl	Cr 121 (29.9) SmA 128 (0.7) I^b,d^
8	**1**	Cr_1_ 51 (−5.1) Cr_2_ 99 (61.6) SmA 148 (1.9) I^c^

^a^The following phases were observed: Cr Crystalline, SmA Smectic A, I Isotropic. Heating rate 10 K min^−1^. ^b^Upon 1st heating. ^c^Data for compounds **8**∙I and **1** were taken from [38]. ^d^Data for compound **7**∙Cl was taken from [42]; heating rate 5 K min^−1^.

**Figure 1:**
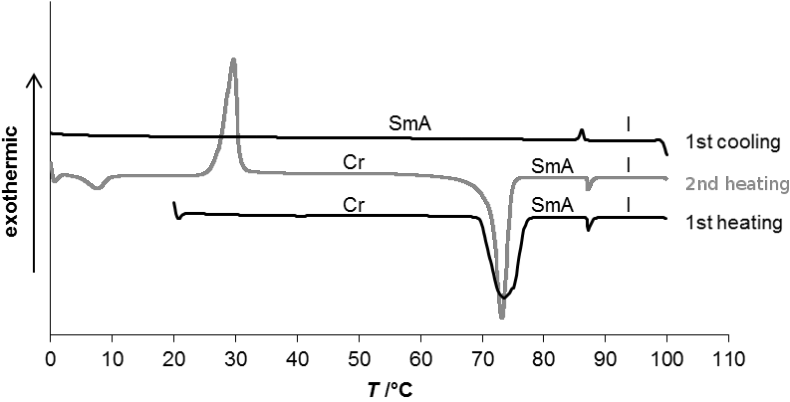
DSC traces of compound **3b** (heating/cooling rate 10 K min^−1^).

N-Methylated guanidinium sulfonimides **2a**,**b** revealed only isotropic melting at 61 °C and 93 °C, respectively (Table 1, entries 1 and 2), while N-methylated guanidinium iodide **8**∙I melted at 117 °C (Table 1, entry 3). In comparison, the N-methylated guanidinium sulfonate **1** displayed a melting transition at 99 °C in the SmA phase and isotropic clearing at 148 °C (Table 1, entry 8). Thus, while the presence of the sulfonimide anions in **2a**,**b** indeed led to a decrease of the melting point as compared to the corresponding iodide **8**∙I, the formation of a smectic mesophase was completely suppressed. For protonated guanidinium sulfonimides **3a**,**b** again a significant decrease of the melting temperature was found (75 °C and 71 °C, respectively, Table 1, entries 4 and 5) as compared to the protonated guanidinium iodide **7**∙I, which melted at 130 °C into the isotropic phase (Table 1, entry 6). The protonated chloride **7**∙Cl showed a melting transition at 121 °C into the SmA phase, and a clearing transition at 128 °C (Table 1, entry 7) [42]. However, while the trifluoromethyl-substituted sulfonimide **3a** displayed only three crystal-to-crystal transitions at 8, 19 and 37 °C, respectively, besides isotropization at 75 °C, the corresponding methyl-substituted sulfonimide **3b** showed two crystal-to-crystal transitions at 5 °C and 27 °C, respectively, a melting transition into the smectic A phase at 71 °C and a clearing point at 87 °C. Thus, sulfonimide-containing ion pair **3b** indeed led to a mesophase albeit with only 16 K phase width as compared to 49 K phase width of the guanidinium sulfonate **1**. The DSC traces of **3b** are shown in Figure 1.

**Figure 2:**
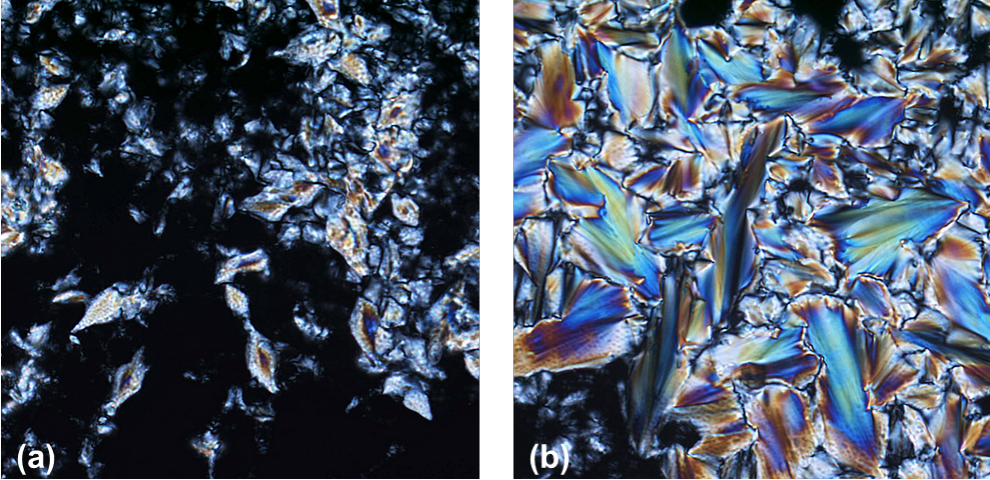
Compound **3b** under crossed polarizers upon cooling from the isotropic melt (200-fold magnification). (a) Homeotropic texture at 80 °C (cooling rate 10 K min^−1^); (b) fan-shaped texture at 88 °C (cooling rate 1 K min^−1^).

POM observations of **3b** upon cooling from the isotropic liquid revealed fan-shaped textures and homeotropic alignment (Figure 2) typical for SmA phases.

**Figure 3:**
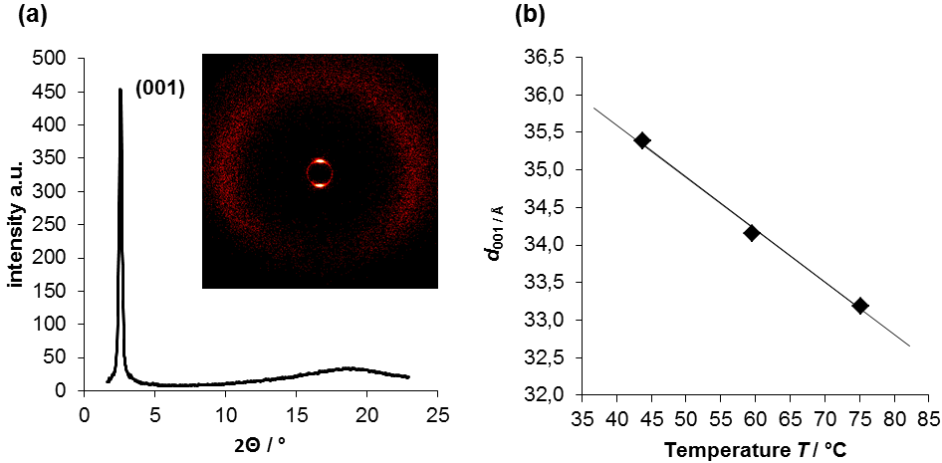
(a) WAXS diffraction pattern of **3b** at 59.5 °C (vertical magnetic field director); (b) temperature-dependent layer spacing of compound **3b**.

The mesophase of compound **3b** was investigated by X-ray scattering (WAXS and SAXS) at different temperatures. The XRD experiments revealed diffraction patterns with a single diffraction peak and a diffuse halo at 4.7 Å resulting from the alkyl chains (Figure 3). These patterns are typical for smectic mesophases and further confirm the assignment of a SmA phase based on POM observations.

The exact layer spacing at each temperature was determined by fitting the first-order peak with a Gaussian distribution (Figure 3 and Supporting Information File 1, Table S1) and decreases with rising temperatures. To allow a comparison with the layer spacings of compounds **1** and **7**∙Cl, the layer spacing of **3b** was determined at a reduced temperature (*T*_red_ = 0.95∙*T*_iso_) by linear extrapolation of the obtained data (Table 2). The obtained value of *d*_red_ = 32.6 Å (Table 2) is in good agreement with the values determined for salts **7**∙Cl (34.0 Å [42]) and **1** (32.2 Å [38]) bearing the same (**7**∙Cl) or nearly the same (N–Me instead of N–H) cation **1**. As the layer spacing is much larger (32.6 Å) compared to the calculated length of the cation and anion (23–24 Å, Table 2), we propose the formation of mixed double layers with the charged heads of cation and anion pointing to each other. This packing behavior is in good agreement with those reported for guanidinium sulfonate **1** [38].

**Table 2:** Layer spacings of compounds **1**, **7**∙Cl and **3b** at a common reduced temperature in comparison to the calculated molecular lengths.

Compound	*T*_red_/[° C]	*d*_red_/[Å]	*L*_calc_ (cation)/[Å]	*L*_calc_ (anion)/[Å]

**3b**	83	32.6	23.8^a^	22.7^a^
**7**∙Cl^b^	122	34.0	23.9	1.81^c^
**1**^d^	141	32.2	23.0	21.0

^a^Calculated using Chem3D Ultra, Cambridgesoft, 2011. ^b^Values were taken from [42]. ^c^Value was taken from [43]. ^d^Values were taken from [38].

## Conclusion

We have developed a route towards guanidinium–sulfonimide ion pairs in which both anion and cation contain mesogenic units. The replacement of a spherical halide counterion by a calamitic sulfonimide anion indeed led to a decrease of the melting points, the effect being larger for trifluorosulfonimides **2a** and **3a** as compared to methylsulfonimides **2b** and **3b**. It should be noted that Strassner has recently introduced a different concept to tune melting points in ionic liquids by electronic effects of the aryl substituent [44–45]. However, the mesogenic sulfonimide resulted in the formation of a SmA mesophase only in the case of **3b**, while ion pairs **2a**,**b** and **3a** did not show any liquid-crystalline properties. Thus, the presence of mesogenic counterions could not overcome the known tendency of sulfonimides to inhibit mesomorphism.

## Experimental

**General Information.** All reactions were carried out under a nitrogen atmosphere with Schlenk-type glassware and the solvents were dried and distilled under nitrogen prior to use. Characterization of the compounds was carried out by using the following instruments. Elemental analyses: Carlo Erba Strumentazione Elemental Analyzer, Modell 1106. NMR: Bruker Avance 500 (^1^H, 500 MHz; ^13^C, 125 MHz). IR: Bruker Vector 22 FTIR spectrometer with MKII golden gate single reflection diamond ATR system. ^1^H and ^13^C NMR spectra were recorded at room temperature and referenced to TMS (Me_4_Si δ_H_ = 0.0 ppm, δ_C_ = 0.0 ppm) as an internal standard. The assignment of the resonances was supported by chemical shift calculations and 2D experiments (COSY and HMBC). MS (EI): Varian MAT 711 spectrometer. Polarizing optical microscopy: Olympus BX50 polarizing microscope combined with a Linkam TP93 central controller. MS (ESI): Bruker Daltonics microTOF-Q spectrometer. Differential scanning calorimetry (DSC): Mettler-Toledo DSC 822e (heating/cooling rates were 10 K min^−1^). X-ray diffraction (WAXS, SAXS regions): Bruker AXS Nanostar C diffractometer employing Ni-filtered Cu Kα radiation (λ = 1.5418 Å). Melting points: Büchi SMP-20. Water content: Metrohm 831 Coulometric Karl Fischer Titrator, (generator electrode with a diaphragm), HYDRANAL-Coulomat AG and HYDRANAL-Coulomat CG solutions were used.

Compounds **4** and **5a,b** are commercially available. Full characterization of compounds **1** and **8**∙I is given in [38], and for compound **7**∙Cl in [42]. For compounds **2b**, **3a** and **7**∙I the following water content was determined by Karl Fischer titration: 0.38%, 0.36% and 0.13%, respectively (see Supporting Information File 1, Table S2).

**4-(Dodecyloxy)-*N*-((trifluoromethyl)sulfonyl)benzenesulfonamide, potassium salt (6a):** A mixture of trifluoromethanesulfonamide (**5a**) (207 mg, 1.38 mmol) and 4-(dodecyloxy)-benzenesulfonylchloride (**4**) (500 mg, 1.39 mmol) was dissolved in abs dichloromethane (20 mL). Abs triethylamine (1 mL, 701 mg, 6.93 mmol) was added and the resulting mixture was heated under reflux for 12 h. After cooling to room temperature the solvent was removed in vacuo, the residue was taken up in ethyl acetate (100 mL), and the hot suspension was filtered. The filtrate was evaporated to dryness and the residue was purified by flash chromatography with ethyl acetate as eluent. The resulting solid was taken up in methanol (20 mL), potassium hydroxide (78 mg, 1.39 mmol) was added, and the mixture was heated under reflux for 5 min. After cooling to 0 °C product **6a** precipitated as a colorless solid. Yield: 425 mg (60%); colorless solid; mp > 300 °C; ^1^H NMR (500 MHz, DMSO-*d*_6_) δ 0.85 (t, *J* = 7.2 Hz, 3H, CH_3_), 1.17–1.35 (m, 16H, CH_2_), 1.36–1.43 (m, 2H, CH_2_), 1.67–1.74 (m, 2H, CH_2_), 4.01 (t, *J* = 6.5 Hz, 2H, OCH_2_), 6.94–7.00 (m, 2H, 3-H), 7.62–7.68 (m, 2H, 2-H) ppm; ^13^C NMR (125 MHz, DMSO-*d*_6_) δ 13.9 (CH_3_), 22.1, 25.4, 28.5, 28.68, 28.71, 28.95, 28.98, 29.0 31.3 (CH_2_), 67.7 (OCH_2_), 113.8 (C-3), 128.1 (C-2), 137.1 (C-1), 160.5 (C-4) ppm; FTIR (ATR) 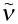: 2917 (m), 2848 (m), 1597 (m), 1584 (m), 1497 (m), 1467 (m), 1387 (w), 1329 (s), 1284 (m), 1254 (m), 1232 (m), 1206 (m), 1160 (vs), 1114 (m), 1093 (s), 1058 (s), 956 (w), 867 (w), 833 (m), 801 (w), 780 (s), 746 (s), 718 (w), 683 (m) cm^−1^; ESIMS (*m*/*z*): 472 [M]^−^, 303 [M^−^ − C_12_H_25_]; HRMS–ESI (*m*/*z*): [M]^−^ calcd for C_19_H_29_F_3_NO_5_S_2_^−^, 472.1445; found, 472.1427.

**4-(Dodecyloxy)-*N*-((methylsulfonyl)benzenesulfonamide, potassium salt (6b):** Methanesulfonamide (**5b**) (277 mg, 2.91 mmol) was given to a suspension of sodium hydride (333 mg, 8.31 mmol) in abs DMF (10 mL) and the mixture was stirred for 1 h. After cooling to 0 °C a solution of 4-(dodecyloxy)benzenesulfonylchloride (**4**, 1.00 g, 2.77 mmol) in abs THF (5 mL) was added dropwise and the reaction mixture was warmed to room temperature. After being stirred for 3 days, the mixture was brought to pH 1 by the addition of concd hydrochloric acid. The solvents were removed in vacuo and the residue was taken up in dichloromethane (50 mL). The resulting solution was dried with magnesium sulfate and filtered, and the filtrate was evaporated to dryness. The residue was taken up in methanol (30 mL) and treated with potassium hydroxide (156 mg, 2.77 mmol) for 5 min. After cooling the mixture to 0 °C the product **6b** precipitated as a colorless solid. Yield: 901 mg (71%); colorless solid; mp > 300 °C; ^1^H NMR (500 MHz, DMSO-*d*_6_) δ 0.86 (t, *J* = 6.8 Hz, 3H, CH_3_), 1.18–1.35 (m, 16H, CH_2_), 1.36–1.43 (m, 2H, CH_2_), 1.66–1.74 (m, 2H, CH_2_), 2.72 (s, 3H, SO_2_CH_3_), 3.98 (t, *J* = 6.5 Hz, 2H, OCH_2_), 6.86–6.92 (m, 2H, 3-H), 7.59–7.64 (m, 2H, 2-H) ppm; ^13^C NMR (125 MHz, DMSO-*d*_6_) δ 13.9 (CH_3_), 22.0, 25.4, 28.5, 28.6, 28.7, 28.90, 28.91, 28.94, 31.2 (CH_2_), 42.6 (SO_2_CH_3_), 67.5 (OCH_2_), 113.2 (C-3), 128.0 (C-2), 138.8 (C-1), 159.6 (C-4) ppm; FTIR (ATR) 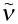: 3077 (w), 2914 (s), 2849 (m), 1595 (m), 1498 (m), 1473 (m), 1394 (m), 1274 (s), 1248 (s), 1152 (s), 1126 (s), 1089 (vs), 972 (m), 831 (s), 808 (s), 721 (s), 679 (m), 590 (s), 525 (vs) cm^−1^; ESIMS (*m*/*z*): 418 [M]^−^, 340 [M^−^ − CH_3_O_2_S + H], 249 [M^−^ − C_12_H_25_]; HRMS–ESI (*m*/*z*): [M]^−^ calcd for C_19_H_32_NO_5_S_2_^–^, 418.1727; found, 418.1728.

### General procedure for the preparation of pentamethylguanidinium ion pairs (**2a,b**)

Potassium carbonate (144 mg, 971 μmol) and methyl iodide (207 mg, 1.46 mmol) were added to a solution of guanidinium chloride (**7**∙Cl, 200 mg, 485 μmol) in acetonitrile (20 mL). The resulting mixture was heated to 50 °C for 12 h and then cooled to room temperature, and the solvent was removed in vacuo. The residue was taken up in dichloromethane (20 mL) and filtered, and the filtrate was concentrated to dryness. A solution of the residue in methanol (20 mL) was treated with silver nitrate (165 mg, 971 μmol) and stirred for 12 h at room temperature under the exclusion of light. The solvent was removed under reduced pressure, the residue was taken up in dichloromethane (20 mL), and the slurry was filtered by using a Rotilabo-syringe filter. After concentration of the filtrate to dryness, the residue was taken up in acetonitrile (10 mL), and sulfonimide salt **6a** or **6b** (509 μmol) was added. The mixture was heated under reflux for 5 min, the solvent was removed in vacuo, and the residue was taken up in dichloromethane (20 mL). After filtration with a Rotilabo-syringe filter the solvent was removed in vacuo, and the crude product was recrystallized from ethyl acetate.

***N*-(4-(Dodecyloxy)phenyl)-*N*,*N*’,*N*’,*N*”,*N*”-pentamethylguanidinium ((4-(dodecyloxy)phenyl)sulfonyl)((trifluoromethyl)sulfonyl)amide (2a):** Yield: 330 mg (79%); colorless solid; ^1^H NMR (500 MHz, CDCl_3_) δ 0.88 (t, *J* = 6.8 Hz, 6H, CH_3_), 1.20–1.38 (m, 32H, CH_2_), 1.40–1.48 (m, 4H, CH_2_), 1.73–1.82 (m, 4H, OCH_2_CH_2_), 2.82, 3.07 (br s, 12H, N[CH_3_]_2_), 3.39 (s, 3H, NCH_3_), 3.94 (t, *J* = 6.5 Hz, 2H, OCH_2_), 3.97 (t, *J* = 6.5 Hz, 2H, OCH_2_), 6.87–6.95 (m, 2H, 3-H, 3”-H), 6.97–7.02 (m, 2H, 2-H), 7.88–7.92 (m, 2H, 2”-H) ppm; ^13^C NMR (125 MHz, CDCl_3_) δ 14.1 (CH_3_), 22.7, 25.98, 26.01, 29.11, 29.18, 29.36, 29.39, 29.40, 29.57, 29.61, 29.64, 29.67, 31.9 (CH_2_), 40.2 (NCH_3_), 40.2, 41.1 (br s, N(CH_3_)_2_), 68.3, 68.5 (OCH_2_), 114.1 (C-3”), 115.9 (C-3), 123.4 (C-2), 129.3 (C-2”), 134.65, 134.72 (C-1, C-1”), 157.7 (C-4), 162.0 (C-4”), 162.2 (C-1’) ppm; ^19^F NMR (235 MHz, CDCl_3_) δ −77.0 (CF_3_) ppm; FTIR (ATR) 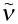: 2918 (s), 2850 (m), 1611 (m), 1597 (m), 1555 (m), 1511 (m), 1473 (m), 1410 (m), 1350 (m), 1323 (s), 1288 (m), 1246 (s), 1221 (m), 1173 (vs), 1132 (s), 1093 (s), 1056 (s), 999 (s), 897 (m), 837 (m), 797 (m), 720 (w), 687 (m) cm^−1^; ESIMS (*m*/*z*): 390 [M]^+^, 222 [M^+^ − C_12_H_25_ + H]; ESIMS (*m*/*z*): 472 [M]^−^, 303 [M^–^ − C_12_H_25_]; HRMS–ESI (*m*/*z*): [M]^+^ calcd for C_24_H_44_N_3_O^+^, 390.3479; found, 390.3456; HRMS–ESI (*m*/*z*): [M]^−^ calcd for C_19_H_29_F_3_NO_5_S_2_^–^, 472.1434; found, 472.1438; DSC: Cr 61 °C [31.8 kJ mol^–1^] I.

***N*-(4-(Dodecyloxy)phenyl)-*N*,*N*’,*N*’,*N*”,*N*”-pentamethylguanidinium ((4-(dodecyloxy)phenyl)sulfonyl)(methylsulfonyl)amide (2b):** Yield: 94 mg (75%); colorless solid; ^1^H NMR (500 MHz, CDCl_3_) δ 0.88 (t, *J* = 6.9 Hz, 6H, CH_3_), 1.20–1.39 (m, 32H, CH_2_), 1.39–1.48 (m, 4H, CH_2_), 1.73–1.81 (m, 4H, OCH_2_CH_2_), 2.82, 3.09 (br s, 12H, N[CH_3_]_2_), 3.41 (s, 3H, NCH_3_), 2.90 (s, 3H, SO_2_CH_3_), 3.90–3.97 (m, 4H, OCH_2_), 6.82–6.87 (m, 2H, 3”-H), 6.89–6.94 (m, 2H, 3-H), 7.00–7.05 (m, 2H, 2-H), 7.87–7.92 (m, 2H, 2”-H) ppm; ^13^C NMR (125 MHz, CDCl_3_) δ 14.1 (CH_3_), 22.7, 26.0, 29.19, 29.20, 29.35, 29.40, 29.58, 29.64, 29.67, 31.9 (CH_2_), 40.3 (NCH_3_), 40.3, 41.2 (br s, N(CH_3_)_2_), 42.2 (SO_2_CH_3_), 68.1, 68.4 (OCH_2_), 113.6 (C-3”), 115.8 (C-3), 123.4 (C-2), 128.7 (C-2”), 135.0 (C-1), 137.8 (C-1”), 157.4 (C-4), 160.7 (C-4”), 162.2 (C-1’) ppm; FTIR (ATR) 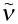: 2919 (s), 2850 (m), 1612 (m), 1552 (m), 1510 (m), 1469 (m), 1403 (m), 1296 (w), 1268 (s), 1241 (s), 1177 (m), 1143 (m), 1122 (s), 1086 (s), 1051 (s), 1001 (w), 948 (w), 897 (w), 837 (m), 801 (m), 721 (s), 653 (s), 587 (vs) cm^−1^; ESIMS (*m*/*z*): 390 [M]^+^, 222 [M^+^ − C_12_H_25_ + H]; ESIMS (*m*/*z*): 418 [M]^−^, 249 [M^−^ − C_12_H_25_]; HRMS–ESI (*m*/*z*): [M]^+^ calcd for C_24_H_44_N_3_O^+^, 390.3479; found, 390.3484; HRMS–ESI (*m*/*z*): [M]^−^ calcd for C_19_H_32_NO_5_S_2_^–^, 418.1716; found, 418.1791; Anal. calcd for C_43_H_76_N_4_O_6_S: C, 63.82; H, 9.47; N, 6.92; found: C, 63.83; H, 9.38; N, 6.94; DSC: Cr 93 °C [59.1 kJ mol^–1^] I.

### General procedure for the preparation of tetramethylguanidinium ion pairs (**3a,b**)

A mixture of guanidinium chloride (**7**∙Cl, 50 mg, 0.122 mmol) and sulfonimide K^+^-salt **6a** or **6b** (0.129 mmol) in acetonitrile (10 mL) was heated under reflux for 5 min. The solvent was removed under reduced pressure, the residue was taken up in dichloromethane (20 mL), and the slurry was filtered with a Rotilabo-syringe filter. After removal of all volatiles in vacuo, the residue was recrystallized from ethyl acetate to give the pure salts **3a**,**b**.

***N*-(4-(Dodecyloxy)phenyl)-*N*’,*N*’,*N*”,*N*”-tetramethylguanidinium ((4-(dodecyloxy)phenyl)sulfonyl)((trifluoromethyl)sulfonyl)amide (3a):** Yield: 95 mg (77%), colorless solid; ^1^H NMR (500 MHz, CDCl_3_) δ 0.88 (t, *J* = 7.0 Hz, 6H, CH_3_), 1.20–1.39 (m, 32H, CH_2_), 1.40–1.49 (m, 4H, CH_2_), 1.73–1.82 (m, 4H, OCH_2_CH_2_), 2.98 (br s, 12H, N(CH_3_)_2_), 3.92 (t, *J* = 6.6 Hz, 2H, OCH_2_), 3.97 (t, *J* = 6.6 Hz, 2H, OCH_2_), 6.85–6.91 (m, 4H, 3-H, 3”-H), 6.95–7.00 (m, 2H, 2-H), 7.85–7.90 (m, 2H, 2”-H) ppm; ^13^C NMR (125 MHz, CDCl_3_) δ 14.1 (CH_3_), 22.7, 25.99, 26.04, 29.13, 29.24, 29.36, 29.39, 29.42, 29.57, 29.59, 29.61, 29.64, 29.67, 31.9 (CH_2_), 40.3 (N(CH_3_)_2_), 66.3, 66.4 (OCH_2_), 113.9 (C-3”), 115.7 (C-3), 122.3 (C-2), 128.7 (C-2”), 130.2 (C-1), 135.6 (C-1”), 157.0 (C-4), 159.1 (C-4”), 161.6 (C-1’) ppm; ^19^F NMR (235 MHz, CDCl_3_) δ −78.1 (CF_3_) ppm; FTIR (ATR) 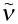: 2917 (s), 2850 (m), 1620 (m), 1595 (m), 1572 (m), 1512 (m), 1474 (m), 1423 (w), 1403 (m), 1335 (s), 1311 (m), 1296 (m), 1257 (m), 1241 (m), 1223 (m), 1195 (s), 1163 (s), 1137 (s), 1111 (w), 1091 (m), 1032 (vs), 915 (w), 828 (s), 782 (m), 752 (m), 723 (m), 685 (m), 640 (m), 598 (s), 562 (s) cm^−1^; ESIMS (*m*/*z*): 376 [M]^+^, 331 [M^+^ − C_2_H_6_N − H]; ESIMS (*m*/*z*): 472 [M]^−^, 303 [M^–^ − C_12_H_25_]; HRMS–ESI (*m*/*z*): [M]^+^ calcd for C_23_H_42_N_3_O^+^, 376.3322; found: 376.3334; HRMS–ESI (*m*/*z*): [M]^−^ calcd for C_19_H_29_F_3_NO_5_S_2_^–^, 472.1434; found, 472.1425; Anal. calcd for C_42_H_71_F_3_N_4_O_6_S_2_ (849.2): C, 59.41; H, 8.43; N, 6.60; found: C, 59.53; H, 8.36; N, 6.60; DSC: Cr_1_ 8 °C [18.6 kJ mol^−1^] Cr_2_ 19 °C [0.8 kJ mol^−1^] Cr_3_ 37 °C [−44.9 kJ mol^−1^] Cr_4_ 75 °C [48.6 kJ mol^−1^] I.

***N*-(4-(Dodecyloxy)phenyl)-*N*’,*N*’,*N*”,*N*”-tetramethylguanidinium ((4-(dodecyloxy)phenyl)sulfonyl)(methylsulfonyl)amide (3b):** Yield: 93 mg (96%); colorless solid; ^1^H NMR (500 MHz, CDCl_3_) δ 0.88 (t, *J* = 6.9 Hz, 6H, CH_3_), 1.20–1.39 (m, 32H, CH_2_), 1.40–1.48 (m, 4H, CH_2_), 1.72–1.81 (m, 4H, OCH_2_CH_2_), 2.86–3.08 (m, 12H, N(CH_3_)_2_), 2.90 (s, 3H, SO_2_CH_3_), 3.92 (t, *J* = 6.5 Hz, 2H, OCH_2_), 3.96 (t, *J* = 6.6 Hz, 2H, OCH_2_), 6.83–6.89 (m, 4H, 3-H, 3”-H), 6.96–7.02 (m, 2H, 2-H), 7.85–7.90 (m, 2H, 2”-H) ppm; ^13^C NMR (125 MHz, CDCl_3_) δ 14.1 (CH_3_), 22.7, 26.0, 26.1, 29.16, 29.25, 29.36, 29.40, 29.42, 29.58, 29.59, 29.61, 29.64, 29.67, 31.9 (CH_2_), 40.4 (N(CH_3_)_2_), 42.4 (SO_2_CH_3_), 66.2, 66.3 (OCH_2_), 113.8 (C-3”), 115.6 (C-3), 122.2 (C-2), 128.7 (C-2”), 130.7 (C-1), 136.7 (C-1”), 156.8 (C-4), 159.2 (C-4”), 161.1 (C-1’) ppm; FTIR (ATR) 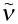: 2915 (s), 2850 (m), 1631 (m), 1597 (m), 1567 (s), 1513 (m), 1467 (m), 1434 (m), 1417 (m), 1401 (m), 1301 (w), 1271 (s), 1239 (s), 1170 (w), 1114 (s), 1079 (vs), 1061 (s), 1004 (m), 972 (m), 913 (w), 835 (s), 800 (m), 714 (s) cm^−1^; ESIMS (*m*/*z*): 376 [M]^+^, 331 [M^+^ − C_2_H_6_N − H]; ESIMS (*m*/*z*): 418 [M]^−^, 249 [M^−^ − C_12_H_25_]; HRMS–ESI (*m*/*z*): [M]^+^ calcd for C_23_H_42_N_3_O^+^, 376.3322; found, 376.3331; HRMS–ESI (*m*/*z*): [M]^−^ calcd for C_19_H_32_NO_5_S_2_^–^, 418.1716; found, 418.1724; Anal. calcd for C_42_H_74_N_4_O_6_S_2_ (795.2): C, 63.44; H, 9.38; N, 7.05; found: C, 63.55; H, 9.31; N, 7.07; DSC: Cr_1_ 5 °C [8.1 kJ mol^−1^] Cr_2_ 27 °C [−54.4 kJ mol^−1^] Cr_3_ 71 °C [73.1 kJ mol^−1^] SmA 87 °C [1.4 kJ mol^−1^] I.

***N*-(4-(Dodecyloxy)phenyl)-*N*’,*N*’,*N*”,*N*”-tetramethylguanidinium iodide (7∙I):** A mixture of guanidinium chloride (**7∙**Cl, 400 mg, 971 μmol) and potassium iodide (493 mg, 2.97 mmol) in acetonitrile (15 mL) was heated under reflux for 5 min. After being cooled to room temperature, the solvent was removed under reduced pressure. The residue was taken up in dichloromethane (20 mL), and the resulting slurry was filtered. After evaporation of the filtrate to dryness the residue was recrystallized from ethyl acetate/acetonitrile (20:1). Yield: 446 mg (94%); colorless solid; ^1^H NMR (500 MHz, CDCl_3_) δ 0.88 (t, *J* = 6.9 Hz, 3H, CH_3_), 1.73–1.80 (m, 2H, OCH_2_C*H*_2_), 2.98 (br s, 12H, N(CH_3_)_2_), 3.91 (t, *J* = 6.6 Hz, 2H, OCH_2_), 6.84–6.91 (m, 2H, 3-H), 7.11–7.17 (m, 2H, 3-H), 9.93 (s, 1H, NH) ppm; ^13^C NMR (125 MHz, CDCl_3_) δ 14.1 (CH_3_), 22.7, 26.0, 29.22, 29.36, 29.41, 29.58, 29.61, 29.64, 29.67, 31.9 (CH_2_), 41.0 (br s, N(CH_3_)_2_), 68.4 (OCH_2_), 115.6 (C-3), 122.6 (C-2), 129.8 (C-1), 157.1, 158.4 (C-4, C-1) ppm; FTIR (ATR) 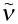: 2917 (s), 2847 (m), 1620 (s), 1558 (s), 1510 (s), 1467 (s), 1452 (m), 1417 (s), 1398 (s), 1312 (m), 1261 (m), 1229 (s), 1167 (m), 1115 (m), 1067 (m), 1024 (m), 1000 (m), 907 (w), 837 (s), 798 (w), 782 (w), 719 (m), 683 (s), 635 (m), 603 (m), 537 (m) cm^−1^; ESIMS (*m*/*z*): 376 [M]^+^, 331 [M^+^ − C_2_H_6_ − H]; ESIMS (*m*/*z*): 127 [M]^−^; HRMS–ESI (*m*/*z*): [M]^+^ calcd for C_23_H_42_N_3_O^+^, 376.3323; found, 376.3343; Anal. calcd for C_23_H_42_IN_3_O (503.5): C, 54.86; H, 8.41; N, 8.35; found: C, 54.91; H, 8.23; N, 7.97; DSC: Cr_1_ 55 °C [−44.3 kJ mol^−1^] Cr_2_ 130 °C [37.2 kJ mol^−1^] I.

## Supporting Information

File 1 DSC traces of compounds **2a**,**b**, **3a** and X-ray data of compound **3b**.
