# Supplementary material for: Synthesis of guanidinium–sulfonimide ion pairs: towards novel ionic liquid crystals
Source: Beilstein J Org Chem. 2013 Jun 5;9:1093–101. doi: 10.3762/bjoc.9.121 (PMC3678594; doi:10.3762/bjoc.9.121)
Supplement: File 1 — DSC traces of compounds 2a,b, 3a and X-ray data of compound 3b. [file Beilstein_J_Org_Chem-09-1093-s001.pdf]

Supporting Information

for

Synthesis of guanidinium–sulfonimide ion pairs: towards novel ionic liquid crystals

Martin Butschies, Manuel M. Neidhardt, Markus Mansueto, Sabine Laschat*, Stefan Tussetschläger

Address: Institut für Organische Chemie, Universität Stuttgart, Pfaffenwalring 55, 70569 Stuttgart, Germany

Email: Sabine Laschat - sabine.laschat@oc.uni-stuttgart.de

*Corresponding author

DSC traces of compounds 2a,b, 3a and X-ray data of compound 3b

DSC traces of compounds (2a,b) and (3a):

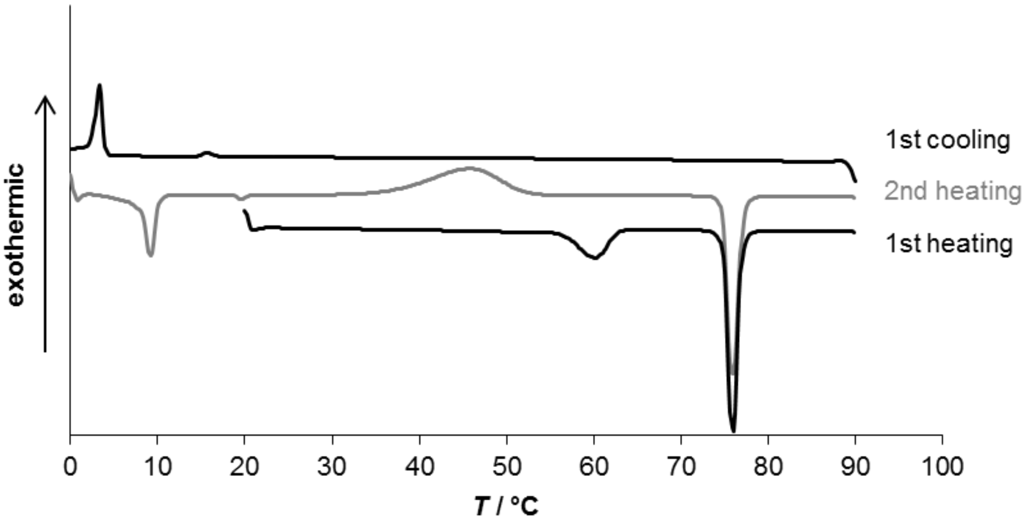

Figure S1: DSC trace of compound **3a** (heating/cooling rate 10 K min^{-1}).

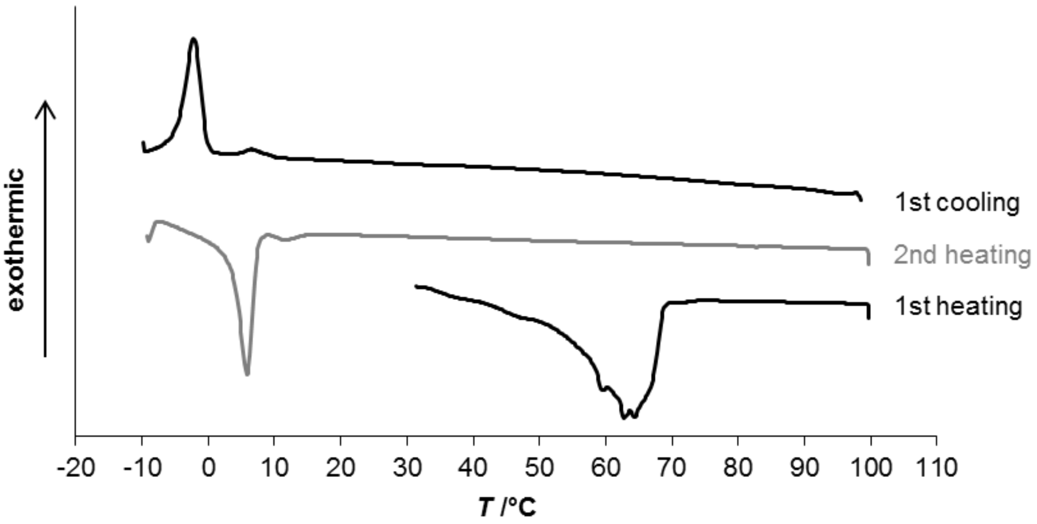

Figure S2: DSC trace of compound **2a** (heating/cooling rate 10 K min^{-1}).

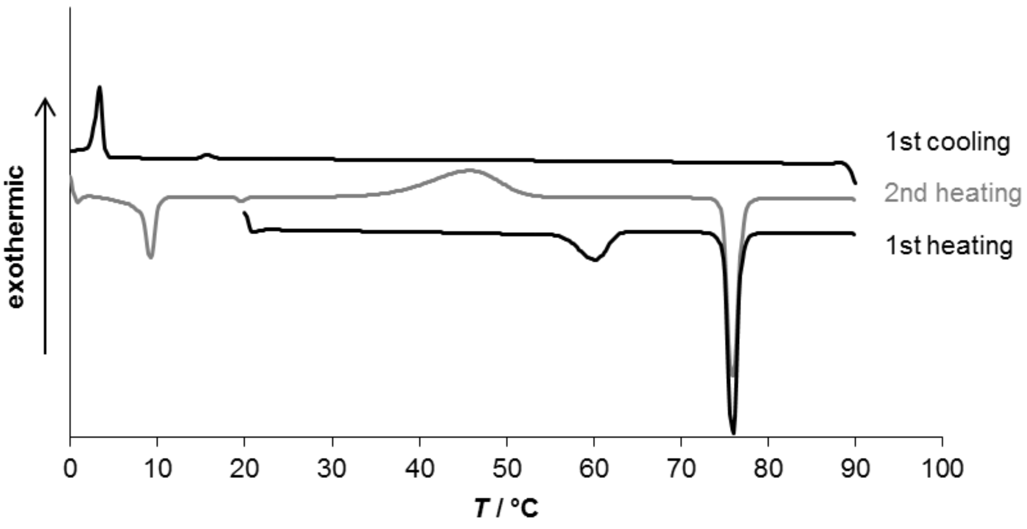

Figure S3: DSC trace of compound **2b** (heating/cooling rate 10 K min^{-1}).

X-ray data of compound (3b):

Table S1: Temperature-dependent layer spacing of compound **3b**.

Temperature T/[°C]	Layer spacing d_{001}/[Å]
75.2	34.19
59.5	34.16
43.8	35.38
WAXS 59.5	4.74 (halo)

Table S2: Water content of selected compounds.

Comp.	Sample weight [mg]	Weight of CH_2Cl_2 [mg]	Concentration (sample weight/ weight of CH_2Cl_2)	Weight of the solution in [mg]	Detected mass of H_2O in [µg]	% H_2O
CH_2Cl_2	1369.8	–	–	1369.8	32.0[(a)]	$2.34 \cdot 10^{-3}$
CH_2Cl_2	1098.9	–	–	1098.9	26.6[(a)]	$2.42 \cdot 10^{-3}$
**7**·I	51.25	2988.71	$1.69 \cdot 10^{-2}$	932.7	42.2	0.13
**2b**	47.38	2076.88	$1.56 \cdot 10^{-2}$	1656.0	136.8	0.38
**3a**	41.45	2606.73	$1.37 \cdot 10^{-2}$	2012.4	145.3	0.36

(a) Ø = 23.8 µg H_2O in 1 g abs. CH_2Cl_2
